# Grandparenting and life satisfaction among Chinese elderlies: a study of possible mechanisms

**DOI:** 10.1186/s12877-023-04540-7

**Published:** 2023-12-11

**Authors:** Xinfeng Cheng, Tolulope Ariyo

**Affiliations:** 1 School of Economics and Management, Xi’an Technological University, Xi’an, 710021 China; 2 School of Health Management, Shangluo University, Shangluo, 726000 China

**Keywords:** Life satisfaction, Older adult, Intergenerational support, Aging attitudes, Grandparenting

## Abstract

**Objective:**

This study examines the impact of caregiving on older people’s life satisfaction, focusing on the role of caring for grandchildren. The study considers individual characteristics (aging attitudes) and situational factors (intergenerational support) and aims to identify the mediating roles of aging attitudes and intergenerational support in the relationship between caregiving and life satisfaction.

**Method:**

The study analyzed data from the 2014 China Longitudinal Aging Social Survey (CLASS), focusing on 5363 grandparents who reported providing care for their grandchildren in the 12 months before the survey. Life satisfaction was subjectively measured. The data was analyzed using multiple linear regression, propensity score matching, and mediation analysis.

**Result:**

The study found that grandparents who cared for their grandchildren have higher levels of life satisfaction. Self-aging attitudes, general aging attitudes, intergenerational economic support, intergenerational instrumental support, and intergenerational emotional support fully mediated the relationship between grandchild care and life satisfaction.

**Conclusion:**

This study demonstrates that caring for grandchildren is a vital activity for older people that helps them develop positive aging attitudes and strengthens intergenerational support, thereby improving their quality of life. Hence, the government, society, families, and communities should provide more social support to older adults caring for grandchildren. This would not only benefit the health of the older people themselves but also promote intergenerational harmony and family development.

## Introduction

Medical science and technology advancements have improved quality of life over the years, leading to an increased life span [1]. As a result, people today are more likely than ever before to live longer and see their third and even fourth generations. This demographic shift not only increases the proportion of older individuals but also changes family structures and caregiving practices [2, 3]. In China, where the older adult population constitutes about 17.8 percent [3], intergenerational support, as influenced by Confucian ideology, means that grandparents often play the role of caregivers to their grandchildren. While several social and economic implications have been attributed to this [4–6], a particular area of interest is how the arrangement tends to impact the life satisfaction of older adults in the context of active aging.

Active aging, which emphasizes maintaining physical, mental, and social activity as individuals age, constitutes a central policy framework for coping with aging across countries worldwide [7]. Likewise, it is becoming a national priority in China as the population ages. The Chinese government's "Opinions on Strengthening Work for the Older People in the New Era" released in November 2021, called for integrating positive aging concepts and healthy aging into the economic and social development process [8]. This strategy aims to enhance older adults' health, social participation, security, and improvement of quality of life as they age.

Quality of life is often measured by perceptions about life satisfaction [9]. As China continues to address population aging, improving the life satisfaction of older people has garnered attention from academic and social circles [10]. This arises from recognizing that various factors affecting older individual's life satisfaction and overall well-being can foster healthy aging, alleviate the strain on social security and familial caregiving, and contribute to both family development and social harmony [11].

Grandchild caregiving involves actively investing time, energy, emotional, economic, and material resources in caring for underage grandchildren [12, 13]. Caring for grandchildren is the most common life event in later life and may either constitute a source of satisfaction or stress for older people [4, 14]. Hence, exploring how caring for grandchildren affects older people’s life satisfaction in the context of an aging population and active aging is crucial.

In Chinese culture, it is common for grandparents to assume the responsibility of caring for their grandchildren, which can effectively alleviate the anxiety associated with parenting and the burden of childrearing [5, 15]. This arrangement can also ease the financial strain on adult children [6]. Despite a significant decrease in families with multiple generations, the proportion of three-generation families (grandparents with adult children and grandchildren) remains significant, highlighting the crucial role of older people in caregiving, families, and society [16]. While previous research underscores the advantage of grandchild care, there is a growing concern regarding its potential adverse effect on the well-being of grandparents [17]. The current body of domestic and international research lacks a consensus regarding the influence of grandchild care on the life satisfaction of older individuals. Additionally, there is a dearth of systematic examinations of the underlying mechanisms that contribute to this impact.

This paper employs data from a comprehensive social survey conducted in China to investigate the association between different forms of caregiving and the life satisfaction of older individuals. Considering the mediating effects of individual characteristics such as aging attitudes and situational factors such as support in grandchild care, the paper investigates the impact of grandparenting on life satisfaction among older Chinese adults.

## Literature review

### Role strain theory and role enhancement theory

Role Strain Theory and Role Enhancement Theory are relevant to exploring relationships between caring for grandchildren and the life satisfaction of older people [17]. The role strain theory posits that when individuals occupy multiple social roles or face conflicting demands within a particular role, they may experience stress, anxiety, and a decreased sense of well-being [18]. These roles may include those related to work, family, community, or other aspects of an individual's life. Based on this theory, older people caring for grandchildren may experience pressure due to conflicting roles, which can become a burden and a source of stress. This can negatively affect their health and well-being, ultimately reducing their overall life satisfaction.

In contrast, Role Enhancement Theory suggests that individuals who successfully fulfill their social roles may experience greater happiness, satisfaction, and well-being. According to this theory, meeting the expectations and demands of one's social roles can provide individuals with a sense of purpose, meaning, and fulfillment in life. Additionally, the theory proposes that the more roles an individual has, the more opportunities they have for social interaction, personal growth, and achievement [19]. Based on the theory’s propositions, when older people take care of their grandchildren, it not only adds to their multiple role experiences but also enhances their sense of value. Additionally, it compensates for the reduction of social networks that often comes with old age, leading to improved social integration and, ultimately, a positive impact on their well-being.

### The effect of caring for grandchildren on life satisfaction among older people

Intergenerational care is a common phenomenon worldwide [20]. Domestically and internationally, researchers have started to focus on the effect of grandparenting on the well-being of older adults, but the findings are mixed [21]. Some studies have reported that caring for grandchildren increases the physical burden [22] and emotional stress [20] in older individuals, resulting in cognitive decline [23]. Additionally, studies have also suggested that grandparents who care for their grandchildren may be unable to participate in other social activities, which lowers their entertainment and leisure time, potentially increasing their risk of depression and decreasing their life satisfaction [24].

On the other hand, some scholars argue that grandparenting can improve the physical and mental health of older adults [25, 26], increase their happiness [27], and extend their life span [28]. A study of 20 European countries found that caring for grandchildren significantly increased the subjective well-being of older adults, regardless of the care intensity [29]. However, studies suggest that the protective effects of caring for grandchildren vary by gender, as older women seem to benefit more than men [30].

### Intergenerational Support as a Mediator

Research on the relationship between grandparenting and life satisfaction in China has explored various mediators, including social support. Chinese cultural norms often dictate strong intergenerational relationships, where older individuals who care for their grandchildren tend to receive increased care and support from their own children [31]. This support can have a profound impact, mitigating the negative effects of stressors related to grandchild care [31, 32]. Empirical evidence has shown that this support can enhance cognitive function, reduce depressive symptoms, and ultimately increase life satisfaction among older grandparents [33, 34].

A recent study that utilized longitudinal data involving 9,219 adults over 45 years [35] showed that caring for grandchildren positively correlates with better mental well-being among Chinese grandparents. Portions of the findings further revealed that children's support significantly mediates the impact of parenting experience on grandparents' mental well-being.

However, despite the existing studies on the mediating role of social support, a literature gap still needs to be addressed. While the literature has recognized the positive effects of intergenerational support, it's important to delve deeper into the specific dimensions of this support (economic, instrumental, emotional) and their differential impact on life satisfaction.

### Aging attitude as a mediator

Aging attitude represents a critical psychosocial factor that can significantly influence an individual's well-being and life satisfaction. Aging attitude refers to an individual's perception of the aging process, and it can be influenced by personal experience and social interaction, including caring for grandchildren [17]. Although a well-established association exists between aging attitudes and well-being/life satisfaction among older adults in China [11, 36], the specific mediating role of aging attitudes between grandparenting and life satisfaction remains understudied with limited evidence.

The role of aging attitudes in mediating the relationship between grandparenting and life satisfaction is vital in Chinese culture, where grandparenting often serves as a means of social integration and expansion of social networks for older individuals [37]. Older individuals who view grandparenting as an opportunity for personal growth, fulfillment, and social connection are more likely to have positive aging attitudes [19]. These positive attitudes may contribute to increased life satisfaction. Conversely, those with negative aging attitudes may perceive grandparenting as a burden or a source of stress, potentially decreasing life satisfaction [20].

In summary, the inclusion of aging attitude and intergenerational support as mediating variables in the relationship between caring for grandchildren and life satisfaction among older Chinese individuals is justified by the need to. These mechanisms not only align with theoretical frameworks related to well-being and aging but also have empirical support from previous studies. By exploring the specific dimensions of intergenerational support and the nuances of aging attitudes, this research aims to provide a deeper and more nuanced understanding of the intricate dynamics involved in how grandparenting impacts the overall life satisfaction of older adults in the Chinese context.

## Data, variables, and methods

### Data

This study analyzed the 2014 Chinese Longitudinal Aging Social Survey (CLASS) data. The CLASS is a large social survey conducted by Renmin University of China, aimed at collecting data on the social and economic conditions, health, family status, and social support of older people aged 60 and above. The project aimed to gain a comprehensive understanding of the various problems and challenges that older adults face during the aging process and provide an important theoretical and factual basis for China to address and solve the problems related to aging.

For the purpose of this study, older adults without minor (underage) grandchildren and those with missing key variables such as aging attitude and intergenerational support were excluded from the sample. The final sample size included for analysis was 5363.

### Variables

#### Dependent variable

Life satisfaction is an individual's subjective assessment of their quality of life [36]. The CLASS questionnaire measures an individual’s life satisfaction with the item "Are you satisfied with your current life?" (Very dissatisfied = 1, relatively dissatisfied = 2, generally = 3, relatively satisfied = 4, very satisfied = 5). This article operationalized this variable as a continuous measure.

#### Independent variable

The study measured the independent variable of grandchild care by asking older people about the time spent caring for their minor grandchildren in the past 12 months. The questionnaire had six response options ranging from every day to rarely or never. The paper considered older adults to be caring for their grandchildren if they cared for at least one minor child. A dummy variable was created with a value of 1 for those who reported caring for their grandchildren and 0 for those who did not. The CLASS questionnaire included questions about five children of the respondents.

#### Mediating variables

This paper considers two mediating variables: aging attitude, which is further divided into self-aging attitude and general aging attitude, and intergenerational support, which is further divided into intergenerational economic support, intergenerational instrumental support, and intergenerational emotional support. The 2014 CLASS questionnaire included seven items from the Attitudes to Aging Questionnaire (AAQ), with a 5-scale rating ranging from "totally disagree" to "fully agree." The titles of these items are as follows: (1) I feel like I am getting old; (2) In my opinion, growing old means losing out on many things; (3) As I age, it becomes harder to make new friends; (4) I feel excluded because of my age; (5) The older one gets, the better they are able to handle life's problems; (6) Wisdom comes with age; (7) There are many enjoyable things about growing old. In this paper, the scores of the first four questions are summed to create a score for “self-aging attitude,” and the scores of the last three questions are summed to create a score for “general aging attitude.” The reliability coefficient for “self-aging attitude” is 0.70, while “general aging attitude” is 0.61.

In this study, the support received through caring for grandchildren was categorized into three dimensions: intergenerational economic support, intergenerational instrumental support, and intergenerational emotional support. Each dimension was assessed using specific topics and response options, as outlined below:

1. Intergenerational economic support: Participants were asked if their child had ever given them money, food, or gifts, and also what it was worth. Response options ranged from Not given = 1; 1–99 yuan = 2; 100–499 yuan = 3; 500–999 yuan = 4; 1000–1999 yuan = 5; 2000–3999 yuan = 6; 4000–6999 yuan = 7; 7000–11999 yuan = 8; and 12,000 yuan or above = 9).
2. Intergenerational instrumental support: Participants were asked to indicate how frequently their child assisted them with household chores in the past 12 months. Response options are as follows: Almost not = 0, several times a year = 1, at least once a month = 2, at least once a week = 3, almost daily = 4).
3. Intergenerational emotional support: Participants were asked to provide a rated response to the question, “Do you think this child does not care enough about you”? Never = 1, occasionally = 2, sometimes = 3, often = 4).

#### Control variables

This research draws on the literature to explore two broad classes of confounding factors [32]: sociodemographic characteristics and health status and residential factors. The first category includes gender, age, marital status, education level, urban or rural residency, personal income, and work status. Marital status is categorized as widowed, divorced, or unmarried (0) and married with a spouse (1). Education level is classified as primary school or below (0) and junior high school or above (1). Household registration attributes are categorized as rural (0) or urban (1). The second category of variables includes health status variables, such as chronic diseases and instrumental mobility of older people, and living pattern variables, such as whether they live alone (living alone = 1; living with others = 0).

### Statistical analysis

The analysis in this paper consists of several steps. First, a multiple linear regression model (OLS) is used to examine the influence of caring for grandchildren on the life satisfaction of the older people. Second, the propensity score matching method (PSM) is employed to test the stability and causality of the relationship between caring for grandchildren and life satisfaction. Finally, mediation analysis is conducted to analyze the mediating roles of aging attitude and intergenerational support in the association between grandchild caregiving and life satisfaction. All analyses were conducted using STATA 12.0 software.

## Results

### Descriptive statistics

Table 1 presents the descriptive statistics of the sample, highlighting key characteristics. The mean age of participants was 67.6 years, and their life satisfaction score averaged 4.07, indicating a level between "more satisfied" and "very satisfied." The self-aging attitude score averaged 11.07, while the general aging attitude score averaged 8.33. Regarding gender distribution, the sample comprised 55.72% men and 44.28% women. Among the participants, 33.88% reported being engaged in grandchild caregiving, while 24% were employed. Furthermore, 75.24% reported having a spouse, and 72% reported having chronic diseases. Approximately 60.86% resided in urban areas; the average instrumental mobility score was 7.73.

**Table 1:** Descriptive statistical results of the samples (*N* = 5363)

Variable name	%	Mean value	Standard deviation	Span
**Dependent variable**				
Life satisfaction		4.07	0.89	1–5
**Independent variable**				
Grandchild care				
yes	33.88			
no	66.12			
**Mediation variable**				
Aging attitude				
Self-aging attitude		11.07	3.93	4–20
General aging attitude		8.33	3.02	3–15
Intergenerational support				
Intergenerational economic support		3.71	1.63	1–6
Intergenerational instrumental support		0.95	1.24	0–4
Intergenerational emotional support		3.64	0.85	1–4
**Controlled variable**				
Age		67.64	6.48	60–113
Sex				
Male	55.72			
Female	44.28			
Marital status				
Have a spouse	75.24			
No spouse	24.76			
Education level				
Primary school and below	57.75			
Junior high school and above	42.25			
Urban and rural attributes				
City	60.86			
Countryside	39.14			
Instrumental Activities of Daily Living (IADL)		7.73	1.75	7–20
Suffer from chronic diseases				
Yes	72.37			
No	27.63			
Residency type				
Living alone	10.22			
Live with others	89.78			
Employed				
Yes	23.83			
No	76.17			
Income		19,514.27	24,514.02	0–960000
Income (logarithm)		9.17	1.60	2.30–13.77

### OLS result

Table 2 shows OLS regression results of the life satisfaction of older people from the entire sample. Model 1 includes control and independent variables, while Model 2 adds aging attitude (self-aging and general aging) and intergenerational support (economic, instrumental, and emotional). Model 1 indicates that caring for grandchildren is significantly and positively associated with life satisfaction (β = 0.060, *p* < 0.05), and the model explains 5.0% of the variance. However, Model 2 shows that self-aging attitude, general aging attitude, and intergenerational support are significantly and positively associated with life satisfaction while caring for grandchildren is not. The model explains 8.4% of the variance. These OLS results suggest that aging attitudes and intergenerational support may mediate the relationship between caring for grandchildren and the life satisfaction of older adults. The model has no multicollinearity (VIF < 2 in both models).

**Table 2:** OLS results on life satisfaction of the older people by caring for grandchildren (*N* = 5363)

	Model 1		Model 2	
Variable	Coefficient	SE	Coefficient	SE
Grandchild care	0.060*	(0.027)	0.020	(0.027)
Age	0.021***	(0.002)	0.022***	(0.002)
Sex	-0.098***	(0.026)	-0.084***	(0.025)
Marital status	-0.024	(0.033)	-0.030	(0.033)
Education level	0.084**	(0.028)	0.057*	(0.027)
Urban and rural attributes	-0.081**	(0.029)	-0.098***	(0.028)
Instrumental Activities of Daily Living (IADL)	-0.057***	(0.007)	-0.046***	(0.007)
Employment status	0.088**	(0.031)	0.096**	(0.031)
Income (logarithm)	0.040***	(0.009)	0.030***	(0.008)
Suffer from chronic diseases	-0.214***	(0.027)	-0.178***	(0.027)
Residency type	0.179***	(0.045)	0.151***	(0.044)
Self-aging attitude			0.014***	(0.003)
General aging attitude			0.030***	(0.004)
Intergenerational economic support			0.036***	(0.007)
Intergenerational instrumental support			0.027**	(0.010)
Intergenerational emotional support			0.100***	(0.014)
Constant	2.751***	(0.167)	1.807***	(0.179)
R-squared	0.050		0.084	

*SE* Standard error

*** *p* < 0.001, ** *p* < 0.01, * *p* < 0.05

### PSM results

This study employed propensity score matching (PSM) to confirm the correlation between caring for grandchildren and the life satisfaction of older people. Three PSM methods were utilized: nearest neighbor, caliper, and kernel matchings. The PSM results (Table 3) showed a good matching effect, and the average treatment effect (ATT) of caring for grandchildren on the life satisfaction of older people was around 0.07–0.08, indicating a significant impact. These results were consistent with the OLS regression outcomes, suggesting that caring for grandchildren was a vital predictor of the life satisfaction of older people. Therefore, it can be concluded that the effect of caring for grandchildren on the life satisfaction of older people was robust.

**Table 3:** Results of ATT estimates of life satisfaction for caring for grandchildren

PSM	Grandparent caregiving	S.E	T
Nearest neighbor matching method	0.078	0.032	2.41
Caliper matching method	0.082	0.029	2.78
Kernel matching method	0.072	0.029	2.51

**Fig. 1:**
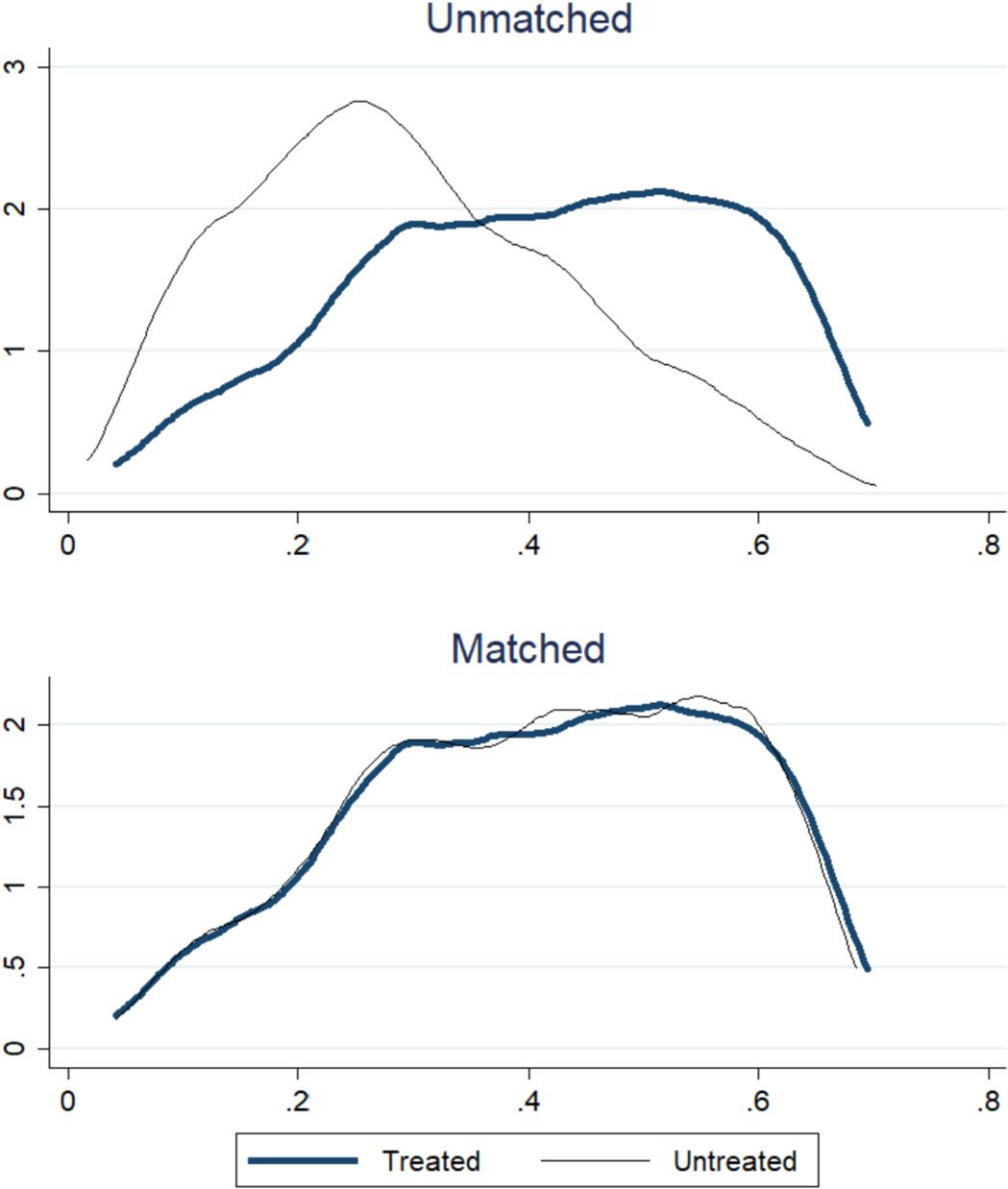
The density comparison before and after matching by the kernel matching method

The analysis of density distribution maps was conducted on both the control and treatment groups before and after employing various propensity score matching (PSM) methods to assess the effectiveness of matching. The results demonstrated a notable reduction in the disparity between the post-treatment and control groups following the matching process, indicating successful matching. Figure 1 visually represents the density comparison before and after matching, utilizing the kernel matching method.

### Mediation analysis

This study examines the mediating effect of aging attitudes and intergenerational support in the full sample. The results of the mediation effect tests are presented in Table 4. The table shows that by including aging attitude and intergenerational support variables, the direct effect of caring for grandchildren on the life satisfaction of older people became insignificant (β = 0.020, *P* = 0.453), while the mediation variables showed a significant mediating role (β = 0.040, *P* = 0.000). Aging attitudes and intergenerational supports completely mediate the relationship between grandchild care and life satisfaction among older adults.

**Table 4:** Results of the mediation effect tests

Effect	Coefficient	SE	P	LLCI	ULCI
Direct effect	0.020	0.027	0.453	-0.033	0.073
Grandchild care → Life satisfaction					
Indirect effect	0.040	0.008	0.000	0.025	0.054
Grandchild care → self-aging attitude → life satisfaction					
Grandchild care → general aging attitude → life satisfaction					
Grandchild care → intergenerational economic support → life satisfaction					
Grandchild care → intergenerational instrumental support → life satisfaction					
Grandchild care → intergenerational emotional support → life satisfaction					

The direct effects of caring for grandchildren on self-aging attitude (β = 0.626, *P* = 0.000), general aging attitude (β = 0.363, *P* = 0.000), economic support (β = 0.114, *P* = 0.020), and instrumental support (β = 0.574, *P* = 0.000) were significant. However, the direct effect on emotional support was not significant (β = -0.002, *P* = 0.953). The direct effects of self-aging attitude (β = 0.014, *P* = 0.000), general aging attitude (β = 0.030, *P* = 0.000), economic support (β = 0.036, *P* = 0.000), instrumental support (β = 0.027, *P* = 0.006), and emotional support (β = 0.100, *P* = 0.000) on life satisfaction were significant.

**Fig. 2:**
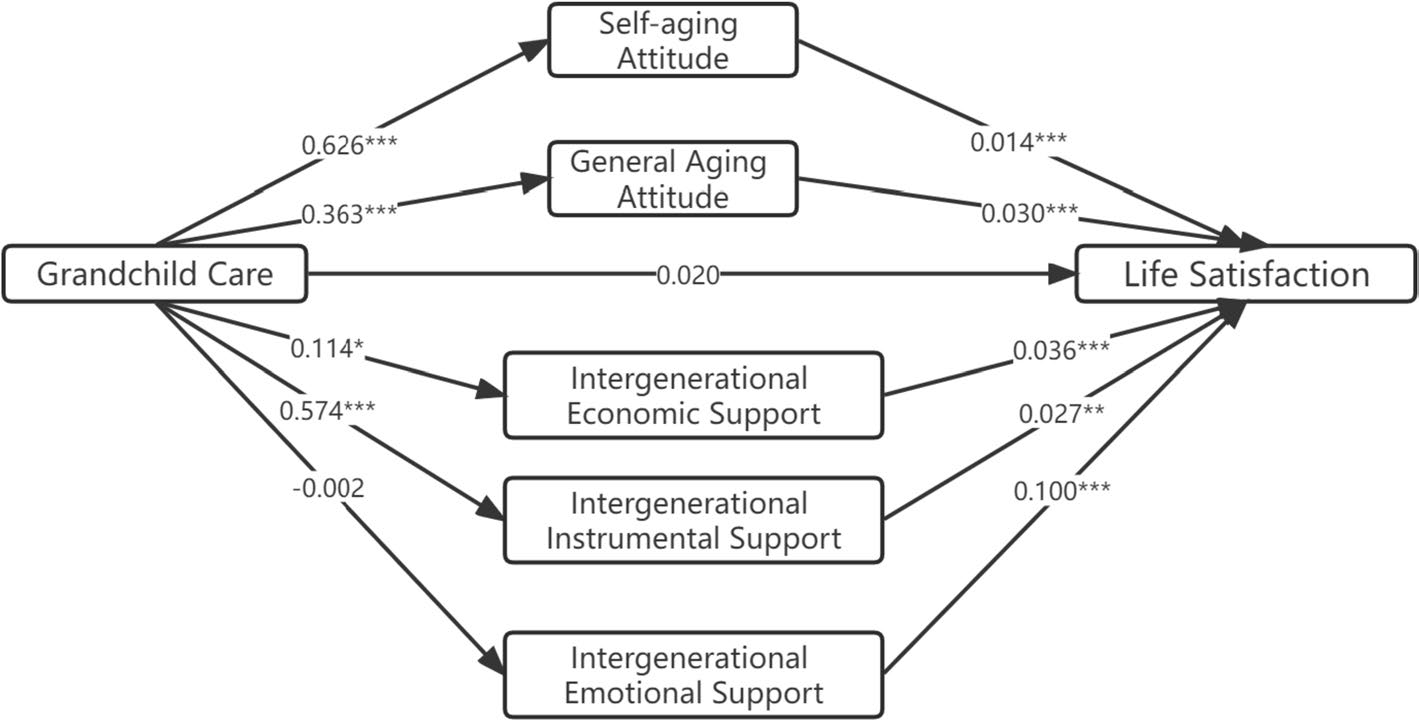
Mechanism of mediation between caring for grandchildren and life satisfaction of older people *** *p* < 0.001, ** *p* < 0.01, * *p* < 0.05

Figure 2 provides a visual representation of the specific mediation effects observed in the study. Path 1 illustrates that caring for grandchildren has a direct effect on self-aging attitude, and this effect mediates a small but significant increase in life satisfaction (mediation size = 0.009). Similarly, Path 2 demonstrates a direct effect of caring for grandchildren on general aging attitude, contributing to increased life satisfaction (mediation size = 0.011). Path 3 indicates that intergenerational economic support has a direct effect on life satisfaction, with a mediation size of 0.004. Path 4 reveals that instrumental support also has a direct effect on life satisfaction, with a mediation size of 0.015. However, path 5 does not show any significant mediation effect of emotional support. Overall, these mediation tests demonstrate that aging attitudes and intergenerational support can play a mediating role in the relationship between caring for grandchildren and life satisfaction.

## Discussion

Using 2014 CLASS data, this study found that caring for grandchildren positively impacts older people's life satisfaction, even after controlling for various factors. The study’s findings provide substantial evidence of the full mediating role of aging attitude and intergenerational support in the said association. The study’s findings, in several parts, are consistent with previous studies, particularly as it concerns the direct association between grandparenting and life satisfaction [26], as well as the mediating role of intergenerational support [12]. However, the finding on the mediating role of aging attitude is unique to the current study and, therefore, contributes to the existing literature and enhances the understanding of the intricate dynamics involved in how caring for grandchildren impacts the overall life satisfaction of older adults.

First, the result on the positive association between grandparenting and life satisfaction among older adults is consistent with Tanskanen’s study conducted with samples of European older adults [26], and supports the role enhancement theory [12]. Caring for grandchildren is an important family participatory activity that reduces the burden on adult children, increases their opportunities, and promotes family happiness [38, 39]. Grandchild care also provides older people with a sense of accomplishment and emotional communication, enhancing their multifaceted roles and a sense of purpose and meaning in life, which improves their life satisfaction. This effect was found to be robust in the PSM test results.

The core aspect of the current study is the investigation of the mechanism of the association, and the study found that caring for grandchildren impacts the life satisfaction of older people through individual and situational factors defined as aging attitudes and intergenerational support. Specifically, grandchild care positively affects the aging attitudes of older people, which is consistent with previous research in the context of China [12]. This significant mediation effect may be attributed to two factors. First, aging is often associated with a shrinking network size [38], the responsibility of grandchild care, however, could provide an avenue for older adults to socialize more and also increase activities of daily living (ADL). Studies have shown a positive association between network size increased ADL and improved life satisfaction among older adults [39, 40]. Secondly, as supported by the role enhancement theory, caring for grandchildren enables older people to realize their value to society and their families, promoting a more positive attitude towards aging and improving their life satisfaction [36].

Furthermore, the finding showed that economic and instrumental support mediates the relationship between grandchild care and life satisfaction among older Chinese adults. Grandparents involved in grandchild care can significantly relieve their adult children of the burden of childcare, allowing them more latitude to focus more on their careers or other personal goals [37]. As a reciprocity gesture, adult children may be inclined to provide more financial and instrumental support to grandparents, strengthening their relationships and contributing to their life satisfaction [41]. This mutual benefit model of Chinese families could play an important role in improving the life satisfaction of older people from the perspective of social exchange theory [38].

Comparatively, the mediated effect size of the general aging attitude was substantially higher than the self-aging attitude. The findings imply that general attitudes about aging are more influential than personal attitudes. It is possible that people are more likely to be influenced by a societal construct or cultural attitude about aging than by their own personal attitude [42]. Also, among the three dimensions of intergenerational support, the result indicated that instrumental support had the most significant mediated effect size. This suggests that instrumental support, which includes financial/material resources and emotional resources, is vital in how older adults perceive life satisfaction [39] even when inundated with the stress of grandparenting.

The findings of this study have significant theoretical and practical implications. Theoretically, this study contributes to the existing literature by revealing the complete mediating role of aging attitudes and intergenerational support in the relationship between caring for grandchildren and the life satisfaction of older individuals, casting light on the complex dynamics at play. Notably, it highlights that society and cultural views on aging might be more influential than personal attitudes and that instrumental assistance, such as financial and emotional resources, is critical in molding older adults' perception of life satisfaction. Practically, these insights underscore the importance of recognizing the social value of older people in supplementing child care and promoting family development. Policymakers and institutions should provide social support to older individuals who care for their grandchildren, encouraging them to maximize family benefits and promote their own healthy aging. Furthermore, older people should recognize their value to their children's family development and cultivate a positive attitude towards aging to achieve healthy aging through social participation. In addition, society should establish a cultural atmosphere that fosters respect and love for older people while rejecting age discrimination.

This study has limitations in several aspects. Firstly, while the 2014 CLASS is relatively old, it was still used for this analysis because the more recent survey waves did not incorporate data on AAQ, an important mediating variable in this study. Though we acknowledge the importance of using more recent data, however, we believe that the dataset remains representative and relevant to our research questions. By using the same dataset for all variables, we maintain data consistency and comparability throughout the analysis. Also, given that China is largely a conservative society, there is likely to be some temporal stability regarding sociocultural and familial dynamics. To this extent, using the 2014 data does not necessarily diminish the relevance of our findings.

Secondly, the cross-sectional data used in this study do not establish causality even with the PSM method. In addition, the study lacks data on how the age difference of the care recipient affects the relationship. Furthermore, caregiving behaviors may affect grandparents' health behaviors, and other mechanisms may be worth exploring. Lastly, the study only examines the relationship between caring for grandchildren and life satisfaction without considering the intensity of care. Despite these limitations, this research makes an additional unique contribution to knowledge regarding the association between caring for grandchildren and life satisfaction. Notably, the study sheds light on the mediating role of the aging attitude, further enhancing our understanding of the complex dynamics involved.

## Conclusion

The study utilized data from the 2014 CLASS to examine how caring for children impacts the life satisfaction of older Chinese adults and the mediating roles of aging attitudes and intergenerational support. The finding showed that caring for grandchildren had a positive impact on older people's life satisfaction. It demonstrated that caring for grandchildren is a vital activity for older people that helps them develop positive aging attitudes and strengthens intergenerational support, thereby improving their quality of life. Encouraging social activities and giving full play to the important value of older people will create an age-friendly society and promote the harmonious development of the aging society. While our study has some limitations, as already acknowledged, future studies are welcome to improve upon it by examining how the duration and intensity of care influence the relationship between grandchild care and life satisfaction.

## Data Availability

The datasets analyzed in the current study is a secondary and publicly accessible data available in the repository of the Institute of Gerontology and National Survey Research Center of Renmin University, China, via http://class.ruc.edu.cn/index.htm
